# Inkjet-Printed Graphene/PEDOT:PSS Temperature Sensors on a Skin-Conformable Polyurethane Substrate

**DOI:** 10.1038/srep35289

**Published:** 2016-10-18

**Authors:** Tiina Vuorinen, Juha Niittynen, Timo Kankkunen, Thomas M. Kraft, Matti Mäntysalo

**Affiliations:** 1 Tampere University of Technology, Department of Electronics and Communications Engineering, Korkeakoulunkatu 3, 33720, Tampere, Finland

## Abstract

Epidermal electronic systems (EESs) are skin-like electronic systems, which can be used to measure several physiological parameters from the skin. This paper presents materials and a simple, straightforward fabrication process for skin-conformable inkjet-printed temperature sensors. Epidermal temperature sensors are already presented in some studies, but they are mainly fabricated using traditional photolithography processes. These traditional fabrication routes have several processing steps and they create a substantial amount of material waste. Hence utilizing printing processes, the EES may become attractive for disposable systems by decreasing the manufacturing costs and reducing the wasted materials. In this study, the sensors are fabricated with inkjet-printed graphene/PEDOT:PSS ink and the printing is done on top of a skin-conformable polyurethane plaster (adhesive bandage). Sensor characterization was conducted both in inert and ambient atmosphere and the graphene/PEDOT:PSS temperature sensors (thermistors) were able reach higher than 0.06% per degree Celsius sensitivity in an optimal environment exhibiting negative temperature dependence.

Vital sign monitoring is evolving from stationary, wire-connected monitoring to a more mobile monitoring with wireless sensor systems. Monitoring devices are shrinking in physical size and weight, and the monitoring electronics are brought closer to the patient, as is already done with wearable measurement devices. Low levels of electrical current drive many physiological functions, and the human body is constantly radiating heat through the skin. Due to these phenomena, several kinds of physiological parameters can be measured using skin mounted devices. One of the interesting parameters is skin temperature, and for that reason a skin thermometer can be utilized in the investigation of cardiovascular health, physical activity and ulcer prediction and prevention^1^,^2^,^3^,^4^. A variety of body monitoring systems are already familiar in both the hospital environment and more casual environments for tracking physical activity. To improve the skin/sensor interface and wearability (comfort and ease of application) in these tracking situations, the development is transitioning from rigid and planar electronic systems towards more adaptable, skin-like electronics^5^,^6^. These types of soft, stretchable, thin-film devices are referred to as epidermal electronic systems (EESs)^7^. EESs are electronic systems which can be placed on human skin, and their structure and mechanical properties mimic the behaviour of the epidermis.

The EES structures need to be very thin and soft to be able to seamlessly integrate with the skin^8^. These properties, however, make the EESs very prone to wrinkling and self-adhesion, or the adhesive may wear down, when they are peeled off from the epidermis. For this reason, the full potential of EESs can be utilized in form of low-cost, disposable epidermal measurements systems^9^. For example, the disposability may be a preferred feature in medical devices where a high level of cleanliness is required. Common fabrication processes for EESs are, for example, spin coating, vacuum deposition of materials, photolithography and etching. However, these can be complex and consume a high degree of materials^7^,^10^,^11^,^12^. Rigid carrier wafers, used in these processes, are incompatible with targeted roll-to-roll (R2R) manufacturing. In addition, photolithography and etching require chemicals and create waste material, and the vacuum deposition of materials is time consuming and has a substrate area limited by the vacuum chamber size^9^. Efficient manufacturing of disposable devices requires simple, material-sparing, and low cost manufacturing processes. Furthermore, exploiting additive printing processes in manufacturing reduces both process steps and waste materials along the EES fabrication process.

To fabricate epidermal electronics, the electronic structures need to withstand the dynamic behaviour and uneven character of the skin. This can be achieved by designing patterns so that the 3D structure of the pattern improves the mechanical properties of the electronic structures. A so-called stretching-patterning-release process, where the substrate is strained prior to material deposition and released afterwards, can be used to pattern stretchable “wavy” patterns^13^. Another possibility is to use in-line horseshoe-like structures which can be found in several applications^7^,^10^,^11^,^12^,^14^. In addition to increasing the stretchability of the 3D structure, the functional material itself can be stretchable. Stretchable, functional materials may be single- or multi-component composites containing functional polymers, nanostructural carbon and metal components^15^,^16^,^17^. In previously mentioned materials the functional component itself may be stretchable, or rigid components are dispersed in a more mechanically deformable matrix. Stretchable, functional materials can also be diluted in solvents, and hence used in form inks. Matsuhisa *et al*. have developed a highly conductive silver ink which consists of silver flakes, fluorine rubber, and fluorine surfactants. Remarkably, with 215% stretching strain the initial conductivity of 738 S cm^−1^ did not decrease lower than 182 S cm^−1 ^18. Bandodkar *et al*. used the conductive polymer poly(3,4-ethylenedioxythiophene):poly(styrenesulfonate) (PEDOT:PSS) and silver/silver chloride (Ag/AgCl) inks where the conductive material was mixed with silicone-based elastomeric material and a non-ionic surfactant^15^.

Conductive polymers are organic polymers, which conduct electricity and may exhibit electrical characteristics that are either like a metal or semiconductor. The widely used PEDOT:PSS has a relatively high conductivity and optical transparency in its doped state^18^. Due to its favourable electrical and optical properties, PEDOT:PSS has been utilized in various applications. This includes, but is not limited to, transparent electrodes for indium tin oxide-free organic light emitting diodes (OLEDs) and polymer solar cells (PSCs), anode material together with ZnO/C hierarchical porous nanorods for lithium ion batteries, and composite electrodes with multi-walled carbon nanotubes for supercapacitors^19^,^20^,^21^,^22^. Moreover, related to this study, PEDOT has also been used for thermal sensors^23^. The temperature dependent behaviour of the PEDOT:PSS originates from the microstructure of the polymer material. PEDOT:PSS forms so-called core-shell structured grains in which the core of the grain is a PEDOT nanocrystal and a PSS-rich shell surrounds the core^24^. The insulating PSS boundaries have a major effect to the overall resistivity of the PEDOT:PSS. At high temperatures the total number of particle boundaries is smaller and the effective “size” of the boundaries is reduced which will reduce the resistance. When the temperature decreases the electrons may not possess enough thermal energy anymore to overcome these boundaries and the resistance increases^25^. In addition to its electrical, thermal and optical properties, PEDOT:PSS has shown interesting mechanical properties to be used in EESs. One such property is its stretchability which is one of the most important mechanical properties for EESs. Lipolmi *et al*. have fabricated transparent conductive films of PEDOT:PSS on a poly(dimethoxysiloxane) (PDMS) substrate, and they have been able to stretch the films up to 188% strain and still retain significant conductivity^26^.

Several kinds of materials and process combinations have been used to fabricate skin-mountable temperature sensors. Furthermore, polymer materials have been used in the fabrication of highly sensitive temperature sensors. Screen printed PEDOT:PSS/CNT temperature sensors were able to reach Temperature Coefficient of Resistance (TCR or α) as high as ~0.61% per degree Celsius^27^. Inkjet-printed temperature sensors, based on carbon and PEDOT:PSS, has been reported to achieve TCR value of 0.25% per degree Celsius^28^. Also other polymers, such as polyaniline nanofibers, have been used to fabricate temperature sensors. Hong *et al*. have been able to manufacture sensors using electrochemical polymerization deposited polyaniline on a PET substrate achieving a 1.0% per degree Celsius sensitivity^29^. Even though these polymer sensors had good sensitivities, they were fabricated on a flexible but not on a stretchable substrate. Temperature sensors have also been fabricated onto stretchable substrates, but by utilizing more conventional fabrication techniques. Chen *et al*. fabricated sputtered Au/Cr temperature sensors on semipermeable stretchable film (Opsite, Smith & Nephew) and the TRC of this device was 0.002778 per degree Celsius^14^. Young *et al*. used a so-called cut-and-paste method with thermal evaporated gold on a PET substrate. The gold pattern was transferred onto the target substrate, which could be a temporary tattoo paper (Silhouette) or a medical tape, such as 3M Tegaderm transparent dressing^9^. However, a new material and manufacturing process combination is required so that the high sensitivity of the polymer material can be combined with a simple fabrication method and still be able to use stretchable substrates.

To simplify the manufacturing process, but at the same time to enable the use of polymer materials with high sensitivities for epidermal electronic systems, we have developed a fabrication method for inkjet-printed graphene/PEDOT:PSS temperature sensors. The choice of the graphene/PEDOT:PSS composite was done based on previous case studies that were performed in our lab that shed light on the electrical properties of the ink and the potential for stretchable applications^30^,^31^. The compatibility of the ink composite (Innophene Phene plus I3015) for inkjet printing was also studied independently by other groups, providing further rationale for the ink’s suitability for our needs^32^. For instance, PEDOT:PSS has already being proven to a have high temperature sensitivity and Honda *et al*. presented in their research that CNT/PEDOT:PSS composition ink had higher sensitivity than CNT or PEDOT:PSS itself^33^. The higher sensitivity was explained to most likely result from electron hopping at the interface of PEDOT:PSS and CNTs. Similar to the role of the CNTs, the graphene flakes in the PEDOT:PSS matrix provides a means for printable, bendable, conductive films^34^.

Our fabrication process utilizes additive printing methods, and thus provides an opportunity to fabricate low-cost and material sparing EESs compatible with disposable devices. In this study, inkjet-printed graphene/PEDOT:PSS temperature sensors with stretchable silver conductors were fabricated on top of a stretchable polyurethane substrate with native adhesive. Just like a bandage, stretchable polyurethane substrates can be used to simulate the mechanical and surface properties of human skin^35^. The main topic is to present a novel fabrication method compatible with stretchable materials but no stretching tests are included in this article. Subsequently, the samples were characterized in ambient and inert atmosphere to analyse their resistance as a function of temperature. Through these measurements, the influence of ambient air was illustrated. Hence, three samples were coated with a fluorochemical acrylic polymer material to prevent, as much as possible, moisture and oxygen from affecting the results. Electrical measurements were repeated after coating to observe the effect of encapsulation.

## Results and Discussion

### Printing process

**Figure 1:**
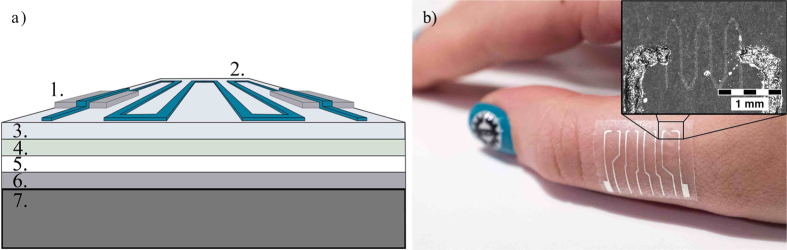
(**a**) Temperature sensor has a multilayered structure of printed functionalities and different bandage layers. Different components are: 1. Screen printed silver conductors, 2. wave patterned graphene/PEDOT:PSS temperature sensor, 3. PU surface layer, 4. adhesive layer, 5. protective paper, 6. PET film and 7. cooling/heating element. (**b**) Photograph of the sample, with sensor array of four sensors, being attached to the skin.

Figure 1(a) shows the final multilayered structure of the printed temperature sensors. The topmost layers are the printed functional structures with screen printed silver conductors and inkjet-printed graphene/PEDOT:PSS temperature sensors. The functional layers were printed on top of an adhesive bandage (plaster), which had polyurethane (PU) surface and polyacrylate adhesive layer. The bottom layer of the plaster was a protective paper, which prevents the adhesive layer from adhering before it is required. The plaster, with printed functionalities, was then placed on top of a polyethylene terephthalate (PET) sheet which provided good mechanical support so that damage during handling was reduced. Figure 1(b) shows the final device attached to the skin.

**Figure 2:**
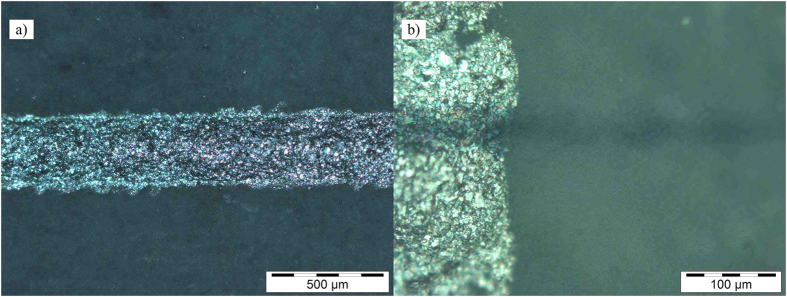
Optical microscope pictures of (**a**) printed silver conductor and (**b**) printed graphene/PEDOT:PSS (horizontal) line crossing the printed silver conductor (vertical line).

Figure 2(a) presents an optical microscope picture of the stretchable silver conductor, whose conductivity under strain was evaluated in a recent study^36^. The line width for the silver conductor was approximately 320 μm. The cross section of the screen printed silver conductor (vertical line) and the inkjet-printed graphene/PEDOT:PSS sensor can be seen in Fig. 2(b). The width of the graphene/PEDOT:PSS sensor was approximately 32 μm and the wetting on top of the polyurethane surface was fairly good. Printing the sensors were done so that the long lines of the sensors were formed in cross-process direction to give some time for the droplets to dry before the next sweep. The printing stability and repeatability of the graphene/PEDOT:PSS ink was good as long as the printing was done continuously. Printing pauses tend to clog the print head nozzles and the best printing stability was achieved with new, freshly filled print heads. If clogging occurred, wiping the nozzle plate with ethanol proved to be the best cleaning procedure.

### Electrical characterization

The initial resistance values were 8.7 kΩ, 9.4, kΩ and 9.7 kΩ for the three characterized samples before the temperature cycling measurements. During measurements, the samples were heated from 35 °C to 45 °C and then cooled back down to 35 °C, using a Peltier element. Human skin temperature may vary depending on the skin location, ambient temperature and possible infection caused fever^37^,^38^. The utilized temperature range in the experiments was chosen due to environmental testing constraints whilst being within the possible temperature deviations on top of the human epidermis.

**Figure 3:**
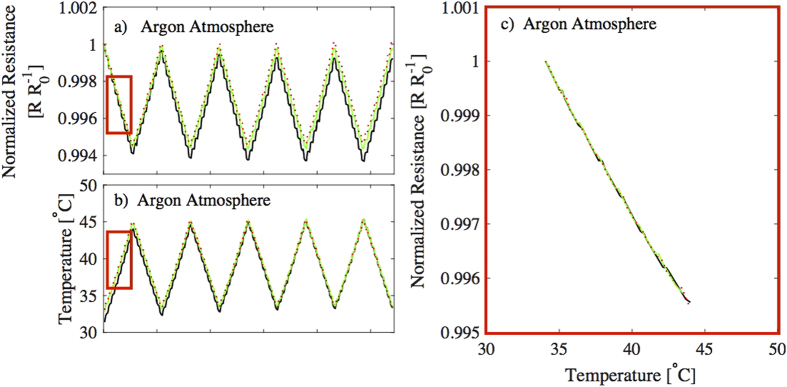
(**a**) normalized resistance and (**b**) temperature from three different samples measured in argon atmosphere. Temperature was increased and decreased between 35 °C and 45 °C along the measurements and this cycling was done five times for each sample. (**c**) The measured data from (**a**,**b**) is transformed in form of normalized resistance as a function of temperature during the first increase from 34 °C to 44 °C.

The temperature cycle was repeated five times for each sample and results from the measurements are presented in Fig. 3. The electrical characterization was conducted in an inert argon atmosphere to minimize the impact of ambient atmosphere factors, such as oxygen or moisture. Figure 3(a) shows normalized resistance and (b) corresponding temperature measurements from three different samples in individual graphs. Resistance decreases when the temperature increases, as was expected according to the other studies done with PEDOT:PSS and graphene, and with other nanostructural carbon/PEDOT:PSS composites, thus graphene/PEDOT:PSS behaves as a negative temperature coefficient (NTC) material^25^,^39^,^40^. Figure 3(c) presents the same results as in Fig. 3(a,b), during the first increase in temperature (from 34 °C to 44 °C), but in this figure, the normalized resistances are shown as a function of temperatures. Those parts of the measurements, where the data is collected for Fig. 3(c), are marked with red rectangles in Fig. 3(a,b). The average TCR of the samples, according to the measurements, is 0.047% per degree Celsius.

**Figure 4:**
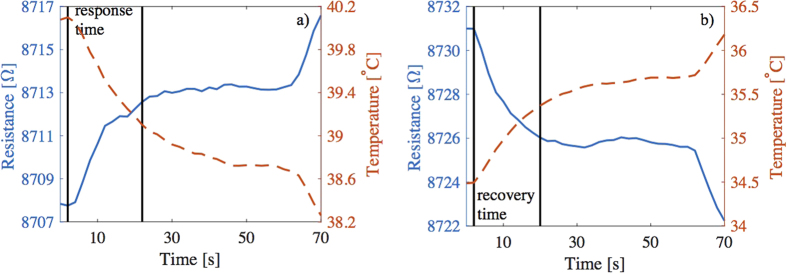
One cooling step (**a**) and one heating step to visualize the response time and the recovery time. The 90 percentile response time was 20 seconds and the 90 percentile recovery time was 18 seconds. The resistance is plotted using a solid line and the temperature is plotted with a dashed line.

Figure 4 presents a response time and a recovery time for one sample. In Fig. 4(a) the input voltage of the Peltier element is decreased by 0.2 V and is then kept unaltered for 1 minute. More detailed description about the measurements can be found from the Methods section. The 90 percentile response time was 20 seconds with this Peltier element setup. It needs to be noticed that the Peltier element also has a response time which reflects to the response time of the temperature sensor. Figure 4(b) shows a 90 percentile recovery time of 18 seconds for the sensor when the Peltier voltage is increased by 0.2 V.

**Figure 5:**
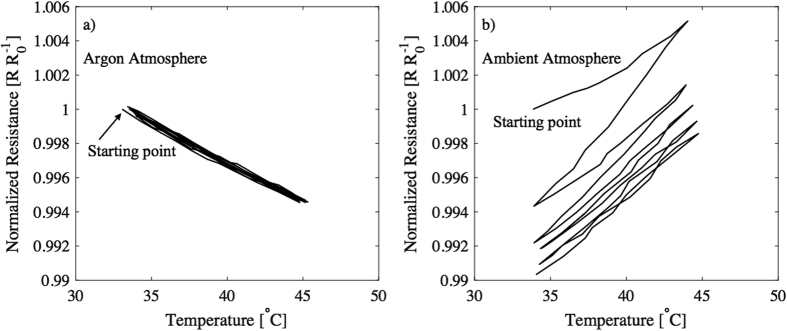
Sample characterized first in (**a**) argon and then moved to (**b**) ambient atmosphere.

Figure 5(a) shows the results from Fig. 3, but now the normalized resistance from one sample is expressed as a function of temperature from all five temperature cycles. Characterization results from all three samples can be found from Supplementary Information (Figure S1). The results show that the temperature sensors exhibit linear NTC behaviour in this temperature range, and the level of hysteresis stays very low. After confirming the functionality in inert atmosphere, the samples were removed from the glove box for additional measurements. Figure 5(b) presents the normalized resistance as a function of temperature in ambient atmosphere. A dramatic divergence can be observed with the non-encapsulated sample’s behaviour relating not only to the slope but also the hysteresis. The slope of the resistance vs. temperature plot turns from negative to positive, which implies that the sensor no longer behaves as an NTC thermistor. This indicates that the device’s temperature sensing ability is skewed by other environmental factors, like humidity. Similarly, humidity has been proven to have an effect on other organic conductors like polyaniline and CNT/PEDOT:PSS nanocomposites^41^,^42^. Likewise, our sensor which utilizes PEDOT:PSS as the conductive matrix and graphene to increase conductivity, is also greatly affected by water vapour. This phenomena was also utilized when Kuberský *et al*. fabricated a humidity sensor using PEDOT:PSS, and they expressed that the change in impedance is based on the dissociation of OH groups in PSS chain and the formation of free charge carriers in PEDOT chain^43^. In fact, it was recently shown that PEDOT:PSS films act as a mixed ionic-electronic conductor and exhibit a variation in its charge transport property as the humidity level is altered^44^,^45^. Furthermore, Nardes *et al*. found that the conductivity of pristine PEDOT:PSS increases by nearly one order of magnitude at 50% relative humidity, as compared to dry films^46^. These studies illustrate that in dry PEDOT:PSS films the charge transport is primarily electronic in nature, whereas the films with elevated water content exhibit an ionic charge transport mechanism. These previous literature results, support the observed effect in our unprotected sample (Fig. 5b) that exhibits a decrease in the organic film’s conductivity as the relative humidity is reduced (via increased temperature). When our samples are removed from the glove box they slowly begin to absorb the ambient water throughout the characterization cycles. As the cycles progress, the overall moisture content of the sample increases and subsequently reduces the overall resistance of the sample. However, even as this secondary effect stabilizes over time, the general trend remains the same; as the temperature is increased there is decreased conductivity as the relative moisture content decreases. This provides further evidence that humidity dramatically affects the PEDOT:PSS/graphene film and that outside the glove box the primary charge transport mechanism is ionic and inside the glove box the dry films charge transport is electronic in nature.

**Figure 6:**
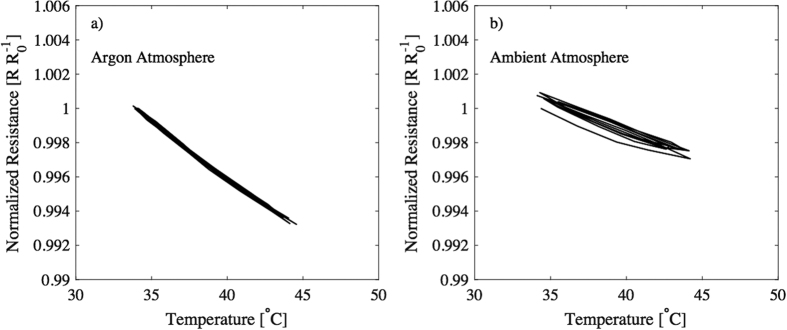
After coating the sample with EGC it was characterized both in (**a**) argon and (**b**) ambient atmosphere.

The results above show that ambient conditions had a severe impact on the results, and for that reason, Novec 1700 Electronic Grade Coating (EGC) coating was used to encapsulate the sensors to reduce the effects of ambient atmosphere. EGC coating provides efficient moisture, chemical and corrosion protection^47^. Three sensors were coated with the EGC and these samples went through the same temperature cycling as in the previous measurements (5 times from 35 °C to 45 °C and back to 35 °C). These characterization results of the coated samples are presented in Fig. 6. Figure 6 shows results only from one sample, and the characterization results from all three samples can be found from Supplementary Information (Figure S2). From Fig. 6(a) it can be seen that the results from measurements done in the inert argon atmosphere after the samples were coated with the EGC. These results illustrated a TRC value of 0.064% per degree Celsius among the samples. Like the previous trials, the samples were then moved from the glove box in an ambient atmosphere and subsequently characterized again (Fig. 6). In this scenario, the sensitivity dropped down to 0.034% per degree Celsius and exhibited an increased hysteresis, however, but the device’s resistance versus temperature was not divergent from the behaviour of the device characterized in a dry environment. This implies that there is a mixed electronic and ionic conductive mechanism, with the former being the primary action of charge transport. Moreover, the reason why the sensors did not function identically in an argon atmosphere and in an ambient air environment, although they were covered with electronic grade coating, is that the coating is only dispensed directly on top of the sensing area. A picture illustrating the covered areas is presented in the Supplementary Information (Figure S3). The ambient atmosphere (i.e. humidity) can still affect the sensors through the bandage material, both from above and underneath the substrate, which is permeable to water and other compounds. The substrate material was chosen so that it would be able to be attached to the skin (medical grade material) and for that reason adding additional coating material on the underside of the substrate was not suitable. In the future, studies to find a more efficient encapsulation to improve the sensor’s behavior under ambient atmospheric conditions will be performed.

## Conclusions

The materials and fabrication processes presented in this paper, enable simple and straightforward fabrication method for inkjet-printed temperature sensors compatible with epidermal electronic systems. Ideally, the printed sensors are targeted for disposable systems, due to the decreased number of fabrication steps and reduced amount of waste material, compared to the traditional lithography processed devices. The sensors were fabricated with inkjet-printed graphene/PEDOT:PSS ink and screen printed silver flake ink. Temperature sensors were printed on top of a skin-formable bandage type substrate, which also provides good adhesion to skin. The completed electronic system is light-weight, thin, and can seamlessly integrate with the skin. The device has the possibility to monitor temperature changes directly on human skin with a TRC value higher than 0.06% per degree Celsius under optimal conditions (35–45 °C). The ambient atmosphere had a severe impact to the results of non-encapsulated devices, nevertheless, the effects not associated with temperature could be reduced using a fluoropolymer coating. The device does not yet compete in efficiency with already existing temperature sensors but the device could be used in its present form as a simple fever indicator on human skin (normal skin temperature about 33 °C). This is due to the simple fabrication process, which enables low-cost fabrication of epidermal electronics with the added value of disposability. Scalable and simple manufacturing process combined with the stretchable, functional materials makes it possible to manufacture epidermal temperature sensors, which are comfortable to use with an excellent skin/device interface.

## Methods

### Substrate preparation

Transparent plasters, adhesive bandages, (Hansaplast) were used as a substrate for the graphene/PEDOT:PSS temperature sensors. These plasters have a transparent PU top layer and an adhesive polyacrylate layer underneath the PU. There is a wound pad, made of PU with hydrocolloid, at the centre of the plasters. This wound pad was cut out from the plasters and only the flat outer sections were used. The plasters were unwrapped from their packaging so that only the supportive film was removed on top of the plaster, and this revealed the outermost PU layer from the plasters. The protective paper underneath the adhesive was kept in place. Pieces of plaster were then attached to Melinex ST506 PET (Teijin DuPont Films) sheet to provide mechanical support during the printing and characterization. This was done to keep the samples unaltered and to prevent possible changes in the structures, and hence in the resistance, due to handling.

### Screen printing process

Silver conductors were screen printed using TIC SCF-300 screen printer and CI-1036 stretchable silver ink (ECM). The silver ink contains 50–60 wt% silver flakes and 1–5 wt% polymer material diluted in a diethylene glycol ethyl ether acetate solvent. Pattern was defined by a polyester screen with a mesh count of 79 threads cm^−1^, mesh opening of 69 μm, and stretching angle of 22,5°. Printed silver conductors were annealed in the convection oven at 130 °C for 13 minutes.

### Inkjet printing and coating process

Temperature sensors were inkjet-printed on the plasters using a Phene Plus I3015 transparent graphene/PEDOT:PSS ink (Innophene). The ink contains 1–5 wt% graphene, the same amount of PEDOT:PSS, and a small amount of diethylene glycol and ethanol (according to the manufacturer). A Dimatix Materials Printer DMP-2831 with a 10 pl drop volume print head was used for the ink deposition. Three layers of graphene/PEDOT:PSS ink with 1270 dpi printing resolution, which equates to 20 μm drop spacing, were printed to fabricate the temperature sensors. The printing process was optimized in a way that the highest possible resistance (to overcome resistance from wires, contacts and other parasitic sources) was achieved with high yield. Three layers with 20 μm drop spacing formed conductive sensors repeatedly. Bringing the layer count down to two decreased the yield significantly and increasing the layer count to four decreased the resistance unnecessarily. Using identical printing parameters for each sample, which all had uneven substrates, caused some variation in sensor resistances. Both the printing plate temperature and the print head temperature were kept at 40 °C. After printing, the sensors were cured in a convection oven at 130 °C for 10 minutes. Three samples were coated with Novec 1700 Electronic Grade Coating (EGC) material (3M). EGC was deposited via pipette on top of the sensors in an argon atmosphere, and subsequently dried at room temperature.

### Electrical characterization setup

To assess the resistance dependence on temperature, a resistance measurement setup was used together with a heating/cooling unit. In the resistance measurement setup, a LabVIEW system design software driven digital multimeter in a VirtualBench (National Instruments) measured the resistance of the samples with a 4.3 Hz sampling rate. VirtualBench also drove the heating/cooling unit, which consisted of a Peltier element and Silverstone Tundra TD03 liquid cooler that kept one side of the Peltier at a constant room temperature. TC-08 Thermocouple Data Logger (Pico Technology) was used to measure the temperature of the plaster surface.

During the electrical characterization of non-encapsulated samples, VirtualBench increased and decreased the Peltier voltage stepwise using 0.2 V steps and the voltage was kept constant for 1 min. For EGC-coated samples the temperature was kept constant for 30 seconds. The temperature cycling fluctuated approximately between 35 °C and 45 °C, and complete cycles (increase from 35 °C to 45 °C and decrease form 45 °C to 35 °C) were done five times for each sample. Electrical measurements were done both in ambient conditions and under inert (argon) atmosphere.

## Additional Information

**How to cite this article**: Vuorinen, T. *et al*. Inkjet-Printed Graphene/PEDOT:PSS Temperature Sensors on a Skin-Conformable Polyurethane Substrate. *Sci. Rep.* **6**, 35289; doi: 10.1038/srep35289 (2016).

## Supplementary Material

Supplementary Information
