# Supplementary material for: Inkjet-Printed Graphene/PEDOT:PSS Temperature Sensors on a Skin-Conformable Polyurethane Substrate
Source: Sci Rep. 2016 Oct 18;6:35289. doi: 10.1038/srep35289 (PMC5082757; doi:10.1038/srep35289)
Supplement: Supplementary Information [file srep35289-s1.pdf]

Inkjet-Printed Graphene/PEDOT:PSS Temperature Sensors on a Stretchable Polyurethane Substrate

Tiina Vuorinen*, Juha Niittynen, Timo Kankkunen, Thomas M. Kraft, Matti Mäntysalo

Supplementary Information

Characterization results from all three different sensors. Results from sample 2 were chosen to be presented more detailed in full paper.

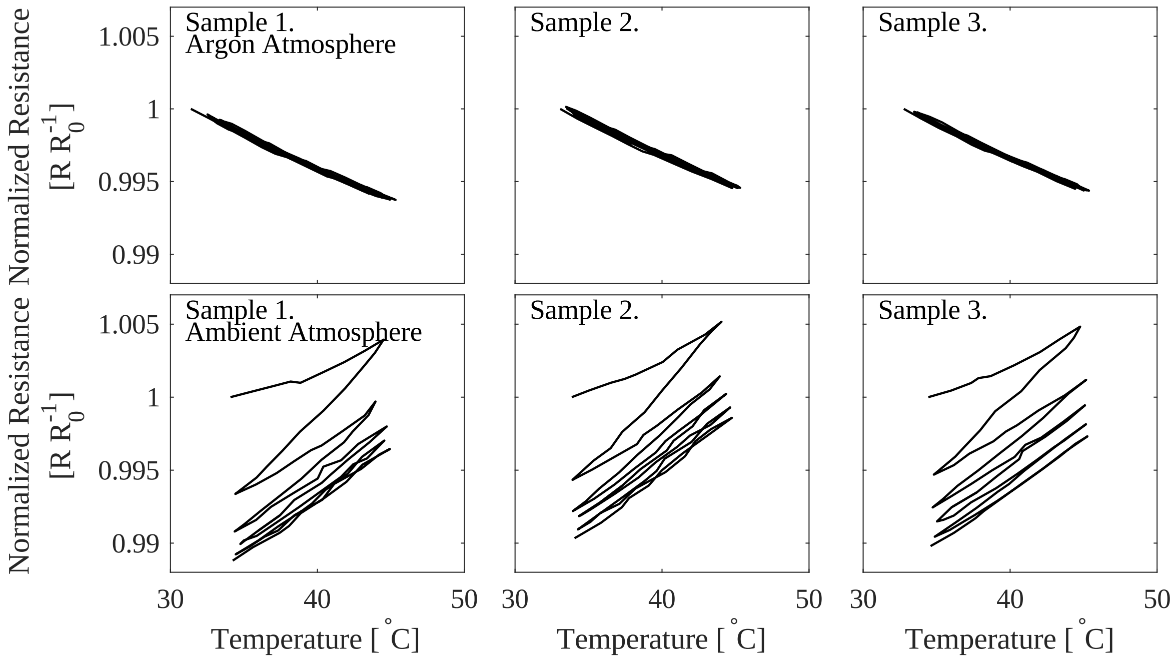

Supplementary Figure S1. Three samples characterized in argon (upper row) and ambient atmosphere (lower row)

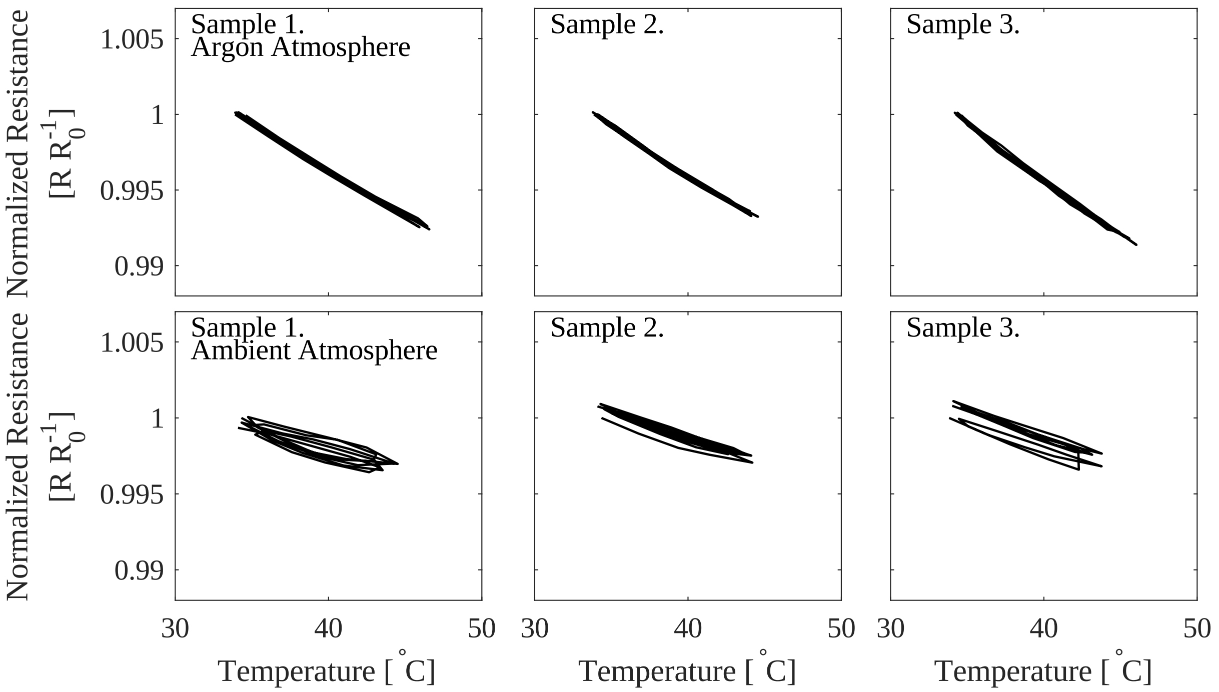

Supplementary Figure S2. Three samples coated with ECG and characterized in argon (upper row) and ambient atmosphere (lower row)

EGC coating material was dispensed using a pipette to cover the sensing areas. Supplementary figure S3 presents the areas covered by the EGC material with blue circles. Numbers and arrows, that can be seen underneath the sensor, are a part of the protective paper, which is removed before the bandage is attached to the skin.

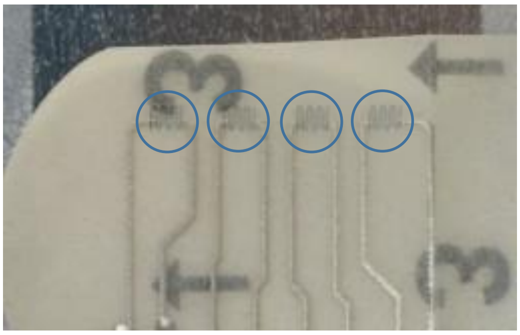

Supplementary Figure S3. Photograph of the sample with EGC covered areas marked with blue circles.
